# Arsenical Paralysis

**Published:** 1882-05

**Authors:** 


### ARSENICAL PARALYSIS.

After describing four cases, Seeligmuller (*Deutsche Medic. Wochenschr.*), comes to the following conclusions: Paralysis may follow either acute or chronic poisoning, the former occurring through the mouth or by absorption through the skin, the latter through the digestive tract or through living in rooms papered with arsenical paper. In acute cases, besides disturbances of the digestive tract, we find severe cerebral disturbances. Severe pains and other disturbances of sensibility occur in the extremities, accompanied by cramps in the flexors, followed by paralysis of the extensors, with atrophy and the reaction of degeneration to the electric current. In chronic poisoning the symptoms of paralysis are less evident, but the disturbances of sensibility more intense. Disturbances of co-ordination may occur which simulate tabes dorsalis. The differential diagnosis between arsenical and saturnine paralysis can be made by the following means:

1. The more rapid inception of arsenical paralysis.
2. The occurrence of alterations of sensibility in arsenical poisoning.
3. The preponderance of paralysis of the upper extremities in saturnine, and of the lower in arsenical poisoning.
4. The more rapid occurrence of muscular atrophy in arsenical poisoning.
5. The presence of trophic changes in arsenical poisoning.
6. The absence of the blue line around the gums.
7. The presence of arsenic in the urine.—*Pacific Medical and Surgical Journal.*

